# Evaluation of Mesh Size in Model Polymer Networks Consisting of Tetra-Arm and Linear Poly(ethylene glycol)s

**DOI:** 10.3390/gels4020050

**Published:** 2018-05-25

**Authors:** Yui Tsuji, Xiang Li, Mitsuhiro Shibayama

**Affiliations:** Institute for Solid State Physics, The University of Tokyo, 5-1-5 Kashiwanoha, Kashiwa 277-8581, Japan; y.tsuji@issp.u-tokyo.ac.jp

**Keywords:** mesh, correlation blob, elastic blob, scaling, model network, tetra-PEG gel

## Abstract

The structure and mechanical properties of model polymer networks consisting of alternating tetra-functional poly(ethylene glycol)s (PEGs) and bis-functional linear PEGs were investigated by dynamic light scattering and rheological measurements. The sizes of the correlation blob ($\xi_{c}$) and the elastic blob ($\xi_{el}$) were obtained from these measurements and compared to the theoretical mesh size, the geometric blob ($\xi_{g}$), calculated by using the tree-like approximation. By fixing the concentration of tetra-PEGs and tuning the molecular weight of linear-PEGs, we systematically compared these blob sizes in two cases: complete network (Case A) and incomplete network (Case B). The correlation blob, $\xi_{c}$, obtained by dynamic light scattering (DLS) was found to obey the well-known concentration dependence for polymer solutions in semidilute regime ($\xi_{c}\sim \phi^{-3/4}$) irrespective of the Cases. On the other hand, the $G^{\prime }$ was strongly dependent on the Cases: For Case A, $G^{\prime }$ was weakly dependent on the molecular weight of linear-PEGs ($G^{\prime }\sim M_{c}{}^{0.69}$) while $G^{\prime }$ for Case B was a strong increasing function of $M_{c}\;$($G^{\prime }\sim M_{c}{}^{1.2}$). However, both of them are different from the geometric blob (theoretical mesh) of the gel networks. In addition, interesting relationships between $G^{\prime }$ and $\xi_{c}$, $G^{\prime }\sim \xi_{c}$, $G^{\prime }\sim \xi_{C}^{-2}$, were obtained for Cases A and B, respectively.

## 1. Introduction

The mesh size is an ambiguous characteristic length in polymer gels. The most intuitive mesh size in a gel for most readers is probably the distance between crosslinkers. Unfortunately, currently, it can only be estimated by theoretical calculation (e.g., tree-like approximation, real space renormalized effective medium approximation) [1,2]. Experimentally, many different sizes have been used as mesh size, including correlation blob ($\xi_{c}$) by scattering experiments [3,4], elastic blob ($\xi_{el}$) by rheological measurements [3,5], and mesh-like structure observed in scanning transmittance electron microscopes (STEM) [6,7].

The images obtained by STEM are probably not the mesh-in, as-prepared gels, especially for those flexible polymer gels (e.g., polyacrylamide gels) because individual polymer chains are too small to be observed, and they usually aggregate with each other to form huge bundles. Instead of direct observation of mesh, the blob concept, introduced by de Gennes, is often used as a measure of mesh-in polymer gels [3]. The de Gennes blob is the “correlation blob” characterized by the correlation length of polymer chains in a crowded system. However, it is misleadingly used to represent the mesh size of polymer networks and gels. A counterexample is a volume-phase transition of poly(*N*-isopropylacryamide) hydrogels [8,9]. By approaching the volume phase transition temperature (≈32 °C), the correlation length diverges while the mesh size diminishes [10,11]. Other than the correlation blob, the elastic blob or tension blob [12] proposed by Pincus is also used as the measure of gel mesh. However, because the general polymer gels are highly heterogeneous, and in general, these inhomogeneities have negative effects on the properties of the gels, such as mechanical properties (fragility and brittleness) [13,14], the comparison between mesh and elastic blob is difficult [7].

**Figure 1:**
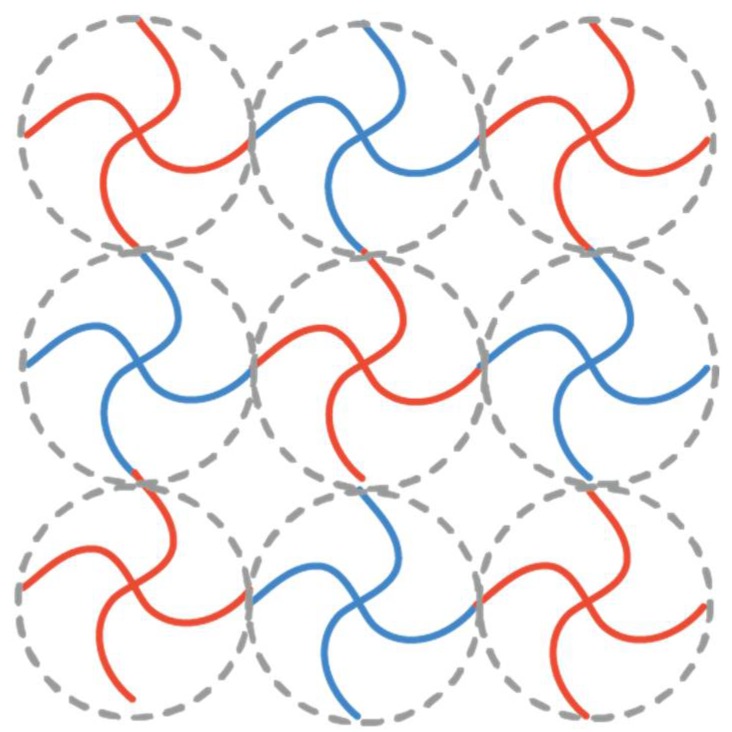
Schematic illustration of polymer networks prepared by mutual reactive tetra-functional poly(ethylene glycol)s (PEG) macromonomers (4 × 4 gels). Blue and red polymer refer to the tetra-functional PEG macromonomers with different end-groups.

In previous studies, Sakai and our groups have reported successful fabrications of nearly-ideal polymer gels overcoming the heterogeneity problems [15,16]. The gels are called tetra-PEG gels, which are formed by cross-end-coupling of two types of tetra-arm poly(ethylene glycol) (tetra-PEG) having complementary end functional groups. Figure 1 shows the schematic illustration of tetra-PEG gels. Tetra-PEG-A (red) and -B (blue) macromers are crosslinked alternatively, forming a three-dimensional infinite polymer network. The elastic modulus of tetra-PEGs is well-described by the phantom network model for $\phi =\phi^{*}$, but it is gradually changed to be represented by the affine network model by increasing $\phi \;\left(\gg \phi^{*}\right)$ [17]. The network structure is described by the Ornstein-Zernike function, $I\left(q\right)=I\left(0\right)/\left(1+\xi_{c}^{2}q^{2}\right)$, irrespective of ϕ as far as the polymer concentration is in the semidilute concentration regime [18]. This means that the structures of tetra-PEG gels are the same as that of polymer solutions in semidilute regime. Here, *I*(*q*) is the scattering intensity, and $\xi_{c}$ is the correlation length (the size of correlation blob). The details of tetra-PEG gels have been described elsewhere [19,20,21,22,23,24,25,26,27,28]. The above-mentioned polymer networks are symmetric polymer networks consisting of tetra-PEGs with equi-molecular weights and equi-functionality. It has been believed that the equi-molecular weight and equi-functionality are the necessary conditions for preparation of “ideal” polymer networks without defects [29,30].

**Figure 2:**
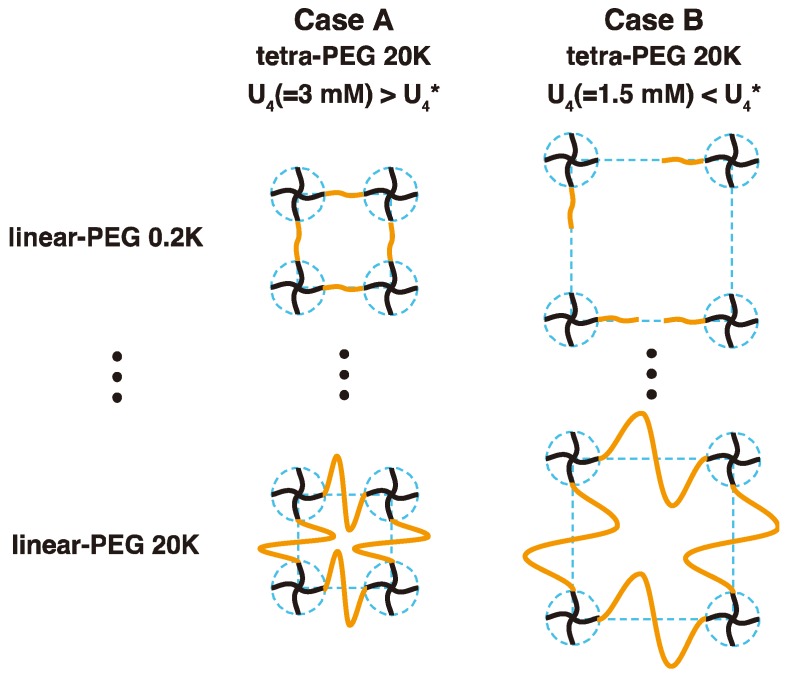
Schematic illustration of 2 × 4 gels formed by mixing mutual reactive tetra-functional PEG and linear-PEG polymers. (**Case A**) Complete network. The molar concentration of tetra-PEG (*U*_4_) in the initial solution is set to be 3.0 mM, higher than its overlapping concentration (*U*_4_^*^ = 2.1 mM for tetra-PEG with molecular weight 20 k) [17]. Linear-PEG with different molecular weights were used as a spacer chain to connect tetra-PEGs. These 2 × 4 gels have the constant crosslinker density but different molecular weights between the crosslinkers. (**Case B**) Incomplete network. *U*_4_ of tetra-PEG in the initial solution is set to be 1.5 mM, lower than its overlapping concentration (*U*_4_^*^ = 2.1 mM). Polymer gels with many defects are expected to be formed for short linear-PEGs and complete network to be formed for long linear-PEGs.

In this work, we have two motivations: (i) The first is a fabrication of “ideal” polymer networks without the above-mentioned constraints. We prepared polymer gels with a combination of tetra-functional PEGs (tetra-PEG) and bis-functional linear PEGs (linear-PEG); hereafter, we call them 2 × 4 gels (Figure 2). We compare their dynamical structure and elastic modulus with those of conventional 4 × 4 gels (Figure 1) and demonstrate the 2 × 4 gels is an alternative method to develop near-ideal network; (ii) The second is elucidation of the physical meaning of “mesh size” in polymer gels by taking advantage of the highly tunable network structure of the 2 × 4. In the case of 4 × 4 gels, the crosslinker density changes with the total polymer volume fraction because the gels are synthesized by only tetra-PEG units. In the case of the 2 × 4 gels, on the other hand, we can independently and systematically tune the molecular weight (linear-PEGs) between the crosslinkers (tetra-PEGs) while maintaining the crosslinker density. The geometric blob, the correlation blob, and the elastic blob are estimated from theoretical calculation, dynamic light scattering (DLS) and rheological measurements, respectively, and their physical meaning are discussed in detail.

## 2. Theoretical

### 2.1. Geometric Blob

The most intuitive characteristic size in a gel is probably the average distance between the crosslinkers or branch points. We call this size a geometric blob ($\xi_{g}$). The number density of the geometric blob ($\rho_{g}$) in a gel can be estimated using the tree-like theory [1] or the real space renormalized effective medium approximation (REMA) [31]. Both theories produce a similar result when the gel network is well-developed. According to the tree-like approximation, the number density of crosslinker (μ) (=number density of geometric blob) in a 2 × 4 gels is given as (See Supplementary Materials) [1]

(1) $$\mu =\rho_{g}=N_{A}U_{4}\left[{\left\{\frac{3}{2}-\;{\left(\frac{1}{p^{2}}-\frac{3}{4}\right)}^{\frac{1}{2}}\right\}}^{3}\left\{3\;{\left(\frac{1}{p^{2}}-\frac{3}{4}\right)}^{1/2}-\frac{1}{2}\right\}\right]$$

where $N_{A}$ is Avogadro constant, $U_{4}$ is the molar concentration of the tetra-PEG units and p is the reaction conversion. By assuming a cubic lattice for simplicity, the size of geometric blob is given as:

(2) $$\xi_{g}=\rho_{g}^{-1/3}$$

### 2.2. Elastic Blob

The elastic blob ($\xi_{el}$) is a characteristic size of elastically effective chains. It was originally introduced by Pincus to explain the stretching of single polymer chain [3,12]. The elastic modulus per blob is in an order of $k_{B}T$, where $k_{B}$ is the Boltzmann constant and T is the absolute temperature. In polymer gels, the elastic blob is considered to be equal to the geometric blob; the polymer chains between crosslinkers are the elastically effective chains. The net elastic modulus ($G^{\prime }$) of the gel is written as the product of number density of elastic blob ($\rho_{el}$) and the elastic modulus per blob,

(3) $$G^{\prime }=\rho_{el}k_{B}T$$

The theoretical prediction for $\rho_{el}$ depends on the models (affine network model or phantom network model), but Equation (3) always holds. $\rho_{el}$ can be estimated by Equation (3) with the elastic modulus of the polymer gels. By assuming a cubic lattice for simplicity, the size of elastic blob is given as:

(4) $$\xi_{el}=\rho_{el}^{-1/3}$$

According to the tree-like approximation, the number density of elastically effective chains should be proportional to the number density of crosslinkers [1]. Therefore, the size of elastic blob is proportional to that of the geometric blob.

(5) $$\xi_{el}\approx \xi_{g}$$

### 2.3. Correlation Blob

The correlation blob ($\xi_{c}$) is a characteristic length, inside which there is higher probability to find a monomer from the same polymer chain rather than that from other chains. The correlation length is also called the screening length of excluded volume effect because the excluded volume effect (intramolecular interaction) vanishes quickly for a length scale larger than the correlation blob due to the presence of other polymer chains. For semidilute polymer solution, the correlation blob has a scaling relation with polymer volume fraction as:

(6) $$\xi_{c}≅R_{g}{\left(\frac{\phi }{\phi^{*}}\right)}^{-\frac{\nu }{3\nu -1}}\sim \phi^{-\frac{\nu }{3\nu -1}},\;\left(\phi \geq \phi^{*}\right)$$

where $R_{g}$ is the gyration radius of the polymer chain, $\phi^{*}$ is the overlapping volume fraction of polymer chains, and ν is the Flory exponent, which shows the solvent quality for the polymer chains (good solvent, ν=3/5 ; *θ*-solvent, ν=1/2).

For a dilute polymer solution, the correlation blob is nearly equal to the size of the polymer chain itself:

(7) $$\xi_{c}≅R_{g},\;\left(\phi \leq \phi^{*}\right)$$

The relaxation time ($\tau^{*}$) of correlation blob can be measured by dynamic light scattering (DLS). By assuming the Stokes-Einstein relation on blob dynamics, the correlation blob is given as:

(8) $$\xi_{c}=\tau^{*}q^{2}\frac{k_{B}T}{6\pi \eta_{s}}$$

where q is the magnitude of the scattering vector, and $\eta_{s}$ is the solvent viscosity.

## 3. Results and Discussion

A series of 2 × 4 gels were prepared by mixing mutual reactive tetra-PEGs (*M*_w_ = 20 k) and linear-PEGs (*M*_w_ = 0.2–20 k) at the stoichiometric ratio. By changing the molecular weight of linear-PEGs while fixing the molar concentration of tetra-PEGs ($U_{4}$), we successfully formed a series of polymer gels with the same crosslinker density but different molecular weights between crosslinkers. The sol samples were prepared as controls by using the same tetra-PEGs and linear-PEGs without mutual reactive end-groups. The 4 × 4 gels were also prepared as controls by using mutual reactive tetra-PEGs with *M*_w_ 20 k. The relaxation time ($\tau^{*}$) of correlation blobs was measured by DLS. $\tau^{*}$ was obtained by fitting the first relaxation mode. Partial heterodyne correction was performed for the relaxation time of polymer gels to obtain the true relaxation time in non-ergodic system [13,32,33]. The size of correlation blob, $\xi_{c}$, was calculated with Equation (8).

**Figure 3:**
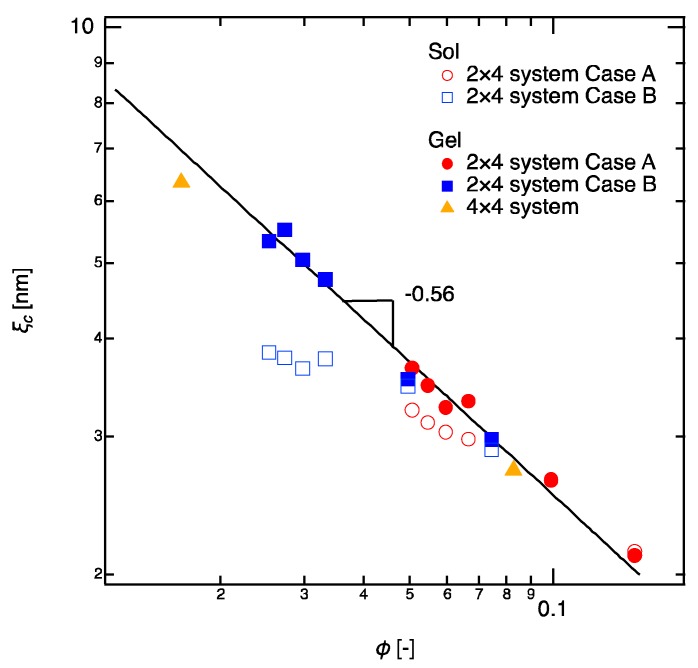
Size of correlation blob in 2 × 4 gels as a function of the total polymer volume fraction ($\phi =\phi_{2}+\phi_{4}$). The full symbols represent the gels and empty symbols represent the sols. Data of Case A are shown in red and those of Case B are shown in blue. Sol samples were prepared as controls by using the non-reactive tetra-PEGs and linear-PEGs. 4 × 4 gels are also shown as controls. The solid line illustrates the fitting curve, $\xi_{c}\sim \phi^{-0.56}$.

In polymer solutions (2 × 4 sols), the scaling law changes from $\xi_{c}\sim \phi^{0}$ to $\xi_{c}\sim \phi^{-0.56}$ as increasing ϕ (Figure 3), corresponding to the general transition of polymer dynamics from dilute region ($\xi_{c}\sim \phi^{0}$) to semidilute region ($\xi_{c}\sim \phi^{-0.75}$ for good solvent and $\xi_{c}\sim \phi^{-1}$ for *θ*-solvent) [34]. The slight deviation of the exponent from that in good solvent is likely due to the strong excluded volume effect of tetra-arm polymers. By contrast, in polymer gels, all the values of $\xi_{c}$ in the gels fall on a single master curve with a scaling law, $\xi_{c}\sim \phi^{-0.56}$ (Figure 3), regardless of the amount of defect (Case A or Case B) and the molecular weight of linear-PEGs. The $\xi_{c}$ of the 4 × 4 gels, which were shown as controls, were on the same master curve of the 2 × 4 gels. The appearance of semidilute scaling law for all the gels suggests that the gels essentially possess the semidilute correlations, which is irrelevant to the network structure. The scaling laws also found by plotting $\xi_{c}$ with respect to molecular weight between crosslinkers ($M_{c}$), but these scaling laws come from the simple relation $\phi \sim U_{4}M_{c}$. Therefore, we do not discuss them here. Another point that we need to emphasize is that the values of $\xi_{c}$ of the gel samples are the same as those of the sol samples when the concentration is high (ϕ>0.04). This result clearly suggests that the correlation blob observed in semidilute regime is independent of the reaction conversion of the polymer chains (or molecular weights) and it is a simple function of concentration as pointed by de Gennes [3].

**Figure 4:**
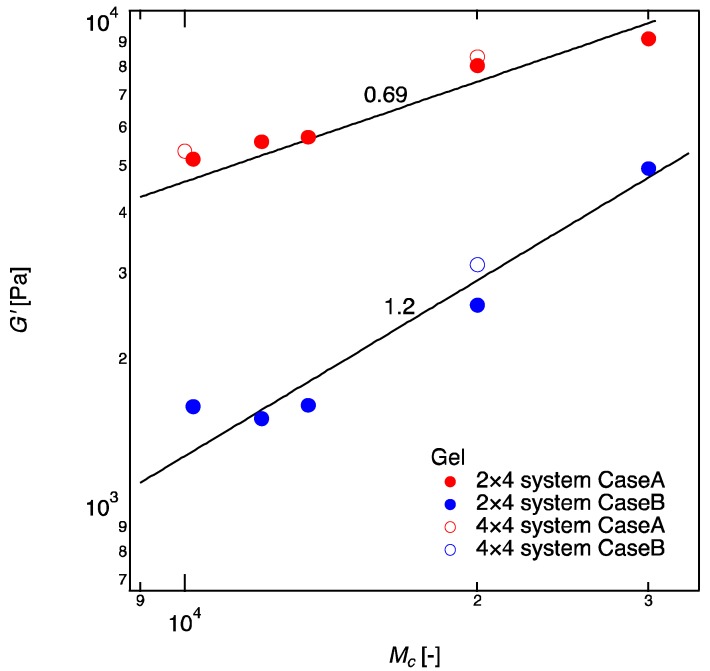
Shear modulus ($G^{\prime }$) of 2 × 4 gels as a function of molecular weight between crosslinkers ($M_{c}$ ). Data of Case A are shown in red and those of Case B are shown in blue. 4 × 4 gels with corresponding tetra-PEG concentration are shown as controls. The solid lines denote the fitting curves of $G^{\prime }\sim M_{c}{}^{0.69}$ and $G^{\prime }\sim M_{c}{}^{1.2}$ for Cases A and B, respectively. The values of $G^{\prime }$ of 4 × 4 gels are cited from the study of Akagi et al. (tetra-PEG 20 k (*M_c_* 10 k), ϕ=0.051 (=3.0 mM) and tetra-PEG 40 k (*M_c_* = 20 k), ϕ=0.096(=3.0 mM) as controls for Case A; tetra-PEG 40 k (*M_c_* = 20 k), ϕ=0.051 (=1.5 mM) as a control for Case B) [17].

Figure 4 shows $G^{\prime }$ of 2 × 4 gels as a function of the molecular weight between crosslinkers ($M_{c}$). $G^{\prime }s$ for the 4 × 4 gels (tetra-PEG gels) with the corresponding crosslinker densities (Case A, *U*_4_ = 3.0 mM; Case B, *U*_4_ = 1.5 mM) and the same $M_{c}$ are shown as controls; the values are cited from the study of Akagi et al. [17]. The values of $G^{\prime }$ were almost the same for 2 × 4 gels and 4 × 4 gels. A simulation and NMR study in 4 × 4 gels has revealed that when the tetra-PEG concentration is above its overlapping concentration (Case A), the unfavorable bonds, such as double-link and higher-order defects, are negligible [35]. Therefore, the comparable values of $G^{\prime }$ in Case A suggest that 2 × 4 gels are free of defects just as the 4 × 4 gels are [15,18,25]. $G^{\prime }$ in Case B increased as the molecular weight of linear-PEGs increased ($G^{\prime }\sim M_{c}{}^{1.2}$), suggesting that more ideal networks are formed when the linear-PEGs are long enough to connect the nearby tetra-PEGs. A precise measurement for the reaction conversion may give us more information to discuss the scaling law in Case B. But it was difficult to measure the reaction conversion in our system at this stage. We could not measure the reaction conversion in Case A as well because the values of $G^{\prime }$ in 2 × 4 gels are very close to those in 4 × 4 gels. Hence, we assume the reaction conversion of 2 × 4 gels in Case A is as high as 4 × 4 gels (reaction conversion ~85% from previous study [17]).

According to the rubber elasticity theory, the elastic modulus is only a function of crosslinker density and does not depend on the molecular weight between crosslinkers [34]. Therefore, theoretically, $G^{\prime }$ in Case A was expected to be constant irrespective of the molecular weight of linear-PEGs, $G^{\prime }\sim M_{c}{}^{0}$. However, from our experiment, we found that the values of $G^{\prime }$ increased with enlarging the molecular weight ($G^{\prime }\sim M_{c}{}^{0.69}$), contracting with the classic theory for rubber elasticity. A similar increase in $G^{\prime }$ was reported by Akagi et al. in 4 × 4 gels, in which they plotted the data as a function of ϕ [17]. They explained this increase as the transition from the phantom network model to the affine network model, and they considered the transition is caused by the suppression of crosslinker fluctuation with increased ϕ. Our data support their prediction and validate the transition more directly because we fixed the crosslinker density and changed the molecular weight between the crosslinkers independently. A big chain placed in between crosslinkers is likely to suppress the motion of the crosslinkers than a small chain.

An interesting experiment was performed by Katashima et al. [36]. Instead of increasing the molecular weight between crosslinkers, they added unattached guest chains into 4 × 4 gels and observed that the concentration of guest chain does not influence the elastic modulus of the gels at all. By comparing their result with ours (Figure 4), we can conclude the fluctuation of crosslinkers is affected by the molecular weight of the crosslinkers but not by the total polymer concentration. This was not clear in previous study by Akagi et al. [17].

**Figure 5:**
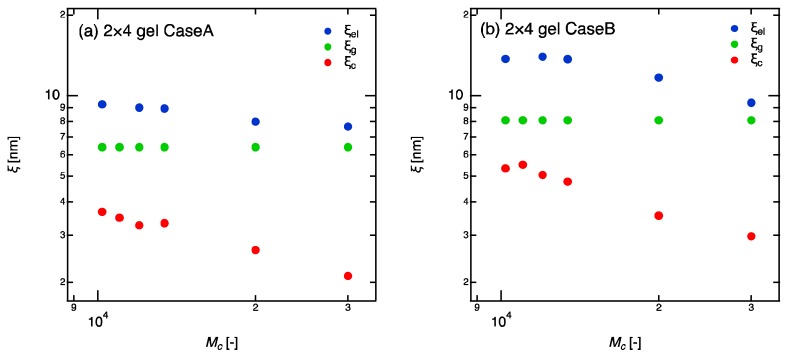
Various blob sizes of 2 × 4 gels as a function of molecular weight between crosslinkers ($M_{c}$). (**a**) Case A: complete network; (**b**) Case B: incomplete network. The values in horizontal axis are the molecular weights of the chain between crosslinkers.

$\xi_{c}$, $\xi_{el}$ and $\xi_{g}$ were estimated from the relaxation time, the elastic modulus, and the tree-like approximation, respectively (Figure 5). In calculation of $\xi_{g}$, we used the Equations (1) and (2) and assumed the reaction conversion, p, is a constant equal to 0.85. $\xi_{g}$ remains constant against $M_{c}$ because the geometric blob, as its definition, does not depend on the molecular weight between crosslinkers. The geometric blob only depends on the crosslinker density (molar concentration of tetra-PEG, $U_{4}$) and reaction conversion. In both Case A and Case B, $\xi_{c}$ were obviously decreasing functions of $M_{c}$ (more accurately, the functions of ϕ because $\phi \sim M_{c}$). This result clearly shows that the correlation blob is definitely not the mesh size in the gels. Although the same conclusion was already reported in the study for volume-phase transition of gels [9], our data shows the correlation blob is not the mesh of gel network even in ordinary as-prepared state. What about the elastic blob, $\xi_{e}$? In Case B, the lack of information for the reaction conversion prevents clear discussion. However, in Case A, the reaction conversion can be estimated to be constant and as high as 85% from the previous studies of 4 × 4 gels [17]. Even in the case, the size of elastic blob decreases with increasing the molecular weight of linear-PEGs, indicating that elastic blob is not a proper method to evaluate mesh size in gels either.

**Figure 6:**
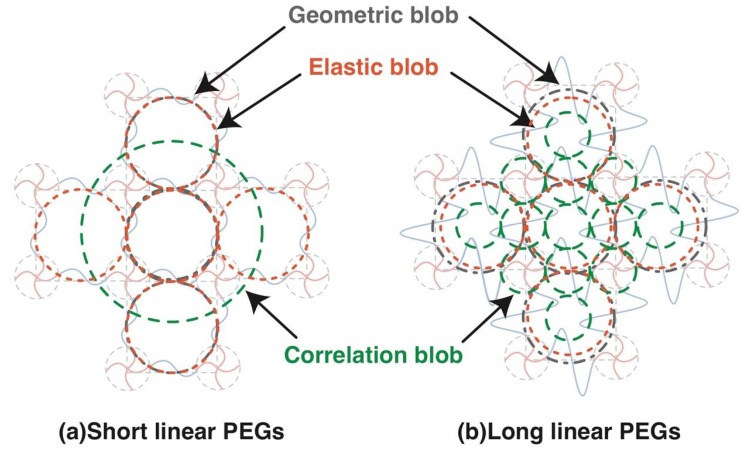
Schematic of various blobs in 2 × 4 gels in Case A complete network. (**a**) Network with short chains between crosslinkers; (**b**) Network with long chains between crosslinkers.

The relation between various blobs in complete network is summarized in Figure 6. While geometric blob and elastic blob do not change with the molecular weight between crosslinkers (or polymer volume fraction), the correlation blob shrinks significantly with increased polymer volume fraction. Although the correlation blob is often referred to as the mesh in many previous studies, it is not the mesh in polymer gels. The elastic blob was expected to be a good measure to estimate the mesh size by experiments, but we found the molecular weight between the crosslinkers strongly influences the estimated values. The evaluation of mesh size in polymer gels is still a challenging task.

**Figure 7:**
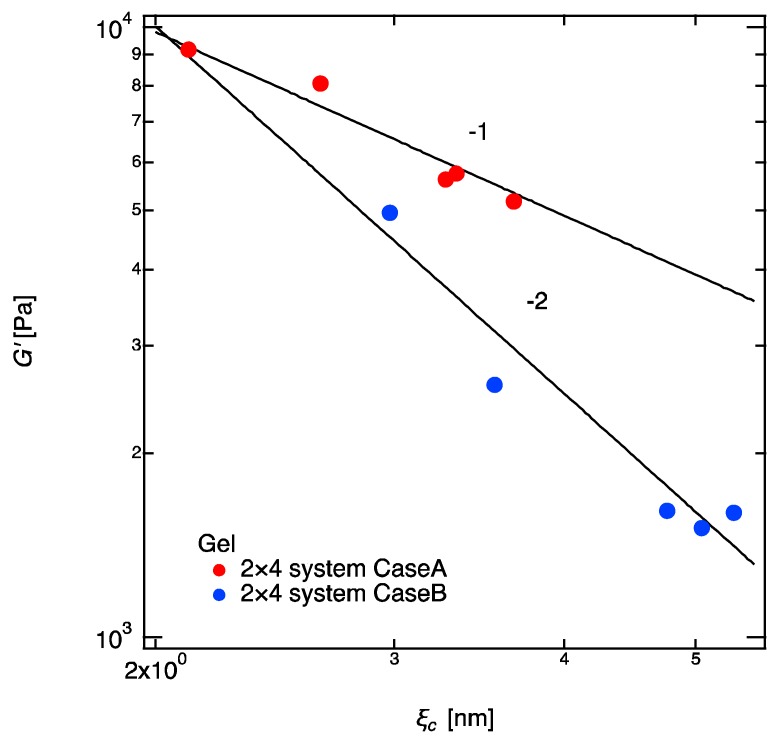
Double logarithmic plot of shear modulus and correlation blob of 2 × 4 gels. The solid lines show the fitting curves of $G^{\prime }\sim \xi_{c}^{-1}$ and $G^{\prime }\sim \xi_{c}^{-2}$ for Cases A and B, respectively.

At the end of this article, we would like to show two interesting new scaling relations between shear modulus $G^{\prime }$ and the correlation blob $\xi_{c}$ (Figure 7): $G^{\prime }\sim \xi_{c}^{-1}$, and $G^{\prime }\sim \xi_{c}^{-2}$ were found in Case A and Case B, respectively. $G^{\prime }$ has been believed to have no correlation with $\xi_{c}$; indeed, the experiment by Katashima et al. proves no correlation between these two parameters when the $\xi_{c}$ is changed by the unattached guest chains (they did not mention this in their article, but their result clearly shows this conclusion) [35]. However, in our experiments, where $\xi_{c}$ is changed by increasing the molecular weight between crosslinkers, $G^{\prime }$ is clearly dependent of $\xi_{c}$. We do not have a confident explanation for the physics lying behind these scaling laws. However, the elastic blobs introduced by Pincus [1,2,3] may be the clue for our findings. According Pincus, the elastic blob size of a single polymer chain is defined as

(9) $$\xi_{el}=\frac{k_{B}T}{f}$$

where f is the stretching force on both ends of the chain. Inside an elastic blob, f is a weak perturbation compared to the thermal energy of the monomers ($k_{B}T$) that randomizes the conformation of polymer chains. Therefore, each elastic blob retains the correlations of a Flory chain (a chain with excluded volume effect). Now, remember that the correlation blob is indeed the screening length of the excluded volume effect. A polymer chain may be divided into elastic blobs as big as correlation blobs, especially when the force is weak. By taking the effect of correlation blob into the classic rubber elasticity theory, we obtain following relation:

(10) $$G^{\prime }=\rho_{el}k_{B}T\sim \nu \xi_{c}^{-1}k_{B}T$$

where ν is the number density of elastically effective chains, $\xi_{c}^{-1}$ denotes the number of correlation blobs per elastically effective chain. Further experimental studies and computer simulations are required to validate this hypothesis.

## 4. Experimental

### 4.1. Sample Preparation

Figure 2 shows the schematic illustration of the model networks, “2 × 4” polymer networks, prepared in this works. The 2 × 4 model networks were prepared by cross-end-coupling of for *N*-hydroxysuccinimide (NHS)-terminated tetra-functional PEG (tetra-PEG, *M*_w_ = 20 k) with amine-terminated linear PEGs (linear-PEGs) having various *M*_w_ (=0.2 k to 20 k) in acetonitrile. The chain-overlap polymer volume fraction ($\phi_{4}^{*}$) of tetra-PEG 20 k is around 0.035 (=2.1 mM) [17]. The subscript 4 denotes tetra-PEG and 2 denotes linear-PEGs hereafter. We started the gel preparation from two cases: Case A, well-packed system ($\phi_{4}=0.050\;\left(=3.0\;\mathrm{mM}\right)>\phi_{4}^{*}$) to form complete networks and Case B, non-packed system ($\phi_{4}=0.025\left(=1.5\;\mathrm{mM}\right)<\phi_{4}^{*}$) to form incomplete networks. The linear-PEGs with different molecular weights were added into the tetra-PEG solutions by the stoichiometric ratio to form polymer gels with the same crosslinker density but different molecular weights between crosslinkers.

For DLS experiment, the corresponding sol samples with the same polymer concentration were prepared by using non-mutual-reactive tetra-PEGs (amine-terminated tetra-PEG) and linear-PEGs (amine-terminated linear-PEG). The 4 × 4 gels were also prepared as controls in DLS experiments by cross-end-coupling of NHS-terminated tetra-PEG (*M*_w_ = 20 k) with amine-terminated tetra-PEG (*M*_w_ = 20 k) equivalently in acetonitrile. We fabricated two gels. One gel forms a complete network ($\phi_{4}=0.083\;\left(=5.0\;\mathrm{mM}\right)>\phi_{4}^{*}$), and the other gel forms an incomplete network ($\phi_{4}=0.017\;\left(=1.0\;\mathrm{mM}\right)<\phi_{4}^{*}$). We note that crosslinker density of 4 × 4 gels is different with 2 × 4 gels here.

### 4.2. Dynamic Light-Scattering Measurements

DLS measurements were performed by ALV5000 Light Scattering Instrument (Langen, Germany). The light source was a He-Ne laser (λ = 632.8 nm), and the scattering angle was 90°. All the experiments were performed at 25 °C. The data was recorded for 30 s for each sample.

### 4.3. Rheological Measurements

The storage modulus (*G*’) of polymer gels were measured with a double cylinder system of a rheometer (MCR501, Anton Paar, Graz, Austria) at 25 °C. The shear strain and the shear frequency were 2% and 1 Hz, respectively.

## 5. Conclusions

We succeeded in preparation of a series of 2 × 4 model polymer networks, where the molecular weights of the linear-PEGs were varied from 0.2 to 20 k. The 2 × 4 gels showed the comparable elastic modulus as the conventional 4 × 4 gels and enabled us independent tuning of the crosslinker density and molecular weight, which was difficult and very limited in 4 × 4 gels. By using the 2 × 4 gels technique, we have access to the huge library of linear polymers with different molecular weights and chemical compositions in the preparation of model networks. Taking the advantage of 2 × 4 gels, we prepared a series of model networks with different molecular weights between the crosslinker while keeping the same crosslinker density, and revisited the old problem, “what is the mesh size in gels?” The following results are disclosed:

(1) The concentration dependence of the correlation length, $\xi_{c}$, is independent of the molecular weight and the completeness of the network structure, and follows the well-known scaling law, $\xi_{c}\sim \phi^{-3/4}$. The gels essentially possess the semidilute correlations, which is irrelevant to the network structure.
(2) In contrast to the correlation length, the mechanical properties, i.e., the elastic modulus, depend strongly on the completeness of the networks, and two different scaling relations were found.
(3) The correlation blob is definitely not the mesh size in polymer gels, although it is often referred to as the mesh size in polymer networks. The elastic blob is, by definition, close to the mesh size. However, it is found that the molecular weight between crosslinkers brings a complicated effect in estimation of the mesh size.
(4) An interesting correlation was found for the first time between $G^{\prime }$ and $\xi_{c}$, depending on the complete/incompleteness of the networks, $G^{\prime }\sim \xi_{c}^{-1}$ and $G^{\prime }\sim \xi_{c}^{-2}$, respectively, for the complete networks and incomplete networks. The Pincus blob may be a clue for explanation of these correlations.

## Supplementary Materials

The following is available online at http://www.mdpi.com/2310-2861/4/2/50/s1.

## References

[1] Miller D.R., Macosko C.W. (1976). A New Derivation of Post Gel Properties of Network Polymers. Macromolecules.

[2] Nishi K., Chijiishi M., Katsumoto Y., Nakao T., Fujii K., Chung U.I., Noguchi H., Sakai T., Shibayama M. (2012). Rubber elasticity for incomplete polymer networks. J. Chem. Phys..

[3] De Gennes P.G. (1979). Scaling Concepts in Polymer Physics.

[4] Oikawa H., Murakami K. (1991). Dynamic light scattering of swollen rubber vulcanizates and the swelling mechanism. Macromolecules.

[5] Sakai T., Kurakazu M., Akagi Y., Shibayama M., Chung U.-I. (2012). Effect of swelling and deswelling on the elasticity of polymer networks in the dilute to semi-dilute region. Soft Matter.

[6] Rüchel R., Brager M.D. (1975). Scanning electron microscopic observations of polyacrylamide gels. Anal. Biochem..

[7] Marmorat C., Arinstein A., Koifman N., Talmon Y., Zussman E., Rafailovich M. (2016). Cryo-Imaging of Hydrogels Supermolecular Structure. Sci. Rep..

[8] Tanaka T. (1978). Collapse of Gels and the Critical Endpoint. Phys. Rev. Lett..

[9] Shibayama M., Tanaka T. (1993). Volume Phase-Transition and Related Phenomena of Polymer Gels. Adv. Polym. Sci..

[10] Tanaka T., Sato E., Hirokawa Y., Hirotsu S., Peetermans J. (1985). Critical kinetics of volume phase transition of gels. Phys. Rev. Lett..

[11] Shibayama M., Tanaka T., Han C.C. (1992). Small angle neutron scattering study on poly(*N*-isopropyl acrylamide) gels near their volume-phase transition temperature. J. Chem. Phys..

[12] Pincus P. (1976). Excluded Volume Effects and Stretched Polymer Chains. Macromolecules.

[13] Shibayama M., Norisuye T., Nomura S. (1996). Cross-link Density Dependence of Spatial Inhomogeneities and Dynamic Fluctuations of Poly(*N*-isopropylacrylamide) Gels. Macromolecules.

[14] Watanabe N., Li X., Shibayama M. (2017). Probe Diffusion during Sol–Gel Transition of a Radical Polymerization System Using Isorefractive Dynamic Light Scattering. Macromolecules.

[15] Sakai T., Matsunaga T., Yamamoto Y., Ito C., Yoshida R., Suzuki S., Sasaki N., Shibayama M., Chung U.-I. (2008). Design and Fabrication of a High-Strength Hydrogel with Ideally Homogeneous Network Structure from Tetrahedron-like Macromonomers. Macromolecules.

[16] Matsunaga T., Asai H., Akagi Y., Sakai T., Chung U.-I., Shibayama M. (2011). SANS Studies on Tetra-PEG Gel under Uniaxial Deformation. Macromolecules.

[17] Akagi Y., Gong J.P., Chung U.-I., Sakai T. (2013). Transition between Phantom and Affine Network Model Observed in Polymer Gels with Controlled Network Structure. Macromolecules.

[18] Matsunaga T., Sakai T., Akagi Y., Chung U.-I., Shibayama M. (2009). SANS and SLS Studies on Tetra-Arm PEG Gels in As-Prepared and Swollen States. Macromolecules.

[19] Kurakazu M., Katashima T., Chijiishi M., Nishi K., Akagi Y., Matsunaga T., Shibayama M., Chung U.-I., Sakai T. (2010). Evaluation of Gelation Kinetics of Tetra-PEG Gel. Macromolecules.

[20] Li X., Tsutsui Y., Matsunaga T., Shibayama M., Chung U.-I., Sakai T. (2011). Precise Control and Prediction of Hydrogel Degradation Behavior. Macromolecules.

[21] Katashima T., Urayama K., Chung U.-I., Sakai T. (2012). Strain energy density function of a near-ideal polymer network estimated by biaxial deformation of Tetra-PEG gel. Soft Matter.

[22] Li X., Khairulina K., Chung U.-I., Sakai T. (2014). Electrophoretic Mobility of Double-Stranded DNA in Polymer Solutions and Gels with Tuned Structures. Macromolecules.

[23] Nishi K., Fujii K., Katsumoto Y., Sakai T., Shibayama M. (2014). Kinetic Aspect on Gelation Mechanism of Tetra-PEG Hydrogel. Macromolecules.

[24] Li X., Kondo S., Chung U.-I., Sakai T. (2014). Degradation Behavior of Polymer Gels Caused by Nonspecific Cleavages of Network Strands. Chem. Mater..

[25] Kamata H., Akagi Y., Kayasuga-Kariya Y., Chung U.I., Sakai T. (2014). “Nonswellable” Hydrogel Without Mechanical Hysteresis. Science.

[26] Kondo S., Hiroi T., Han Y.S., Kim T.H., Shibayama M., Chung U.I., Sakai T. (2015). Reliable Hydrogel with Mechanical “Fuse Link” in an Aqueous Environment. Adv. Mater..

[27] Li X., Watanabe N., Sakai T., Shibayama M. (2017). Probe Diffusion of Sol–Gel Transition in an Isorefractive Polymer Solution. Macromolecules.

[28] Li X., Hirosawa K., Sakai T., Gilbert E.P., Shibayama M. (2017). SANS Study on Critical Polymer Clusters of Tetra-Functional Polymers. Macromolecules.

[29] Kondo S., Chung U.-I., Sakai T. (2013). Effect of prepolymer architecture on the network structure formed by AB-type crosslink-coupling. Polym. J..

[30] Kondo S., Sakurai H., Chung U.-I., Sakai T. (2013). Mechanical Properties of Polymer Gels with Bimodal Distribution in Strand Length. Macromolecules.

[31] Nishi K., Noguchi H., Sakai T., Shibayama M. (2015). Rubber elasticity for percolation network consisting of Gaussian chains. J. Chem. Phys..

[32] Pusey P.N., Van Megen W. (1989). Dynamic light scattering by non-ergodic media. Phys. A Stat. Mech. Appl..

[33] Joosten J.G.H., McCarthy J.L., Pusey P.N. (1991). Dynamic and static light scattering by aqueous polyacrylamide gels. Macromolecules.

[34] Rubinstein M., Colby R.H. (2003). Polymer Physics.

[35] Lange F., Schwenke K., Kurakazu M., Akagi Y., Chung U.-I., Lang M., Sommer J.-U., Sakai T., Saalwächter K. (2011). Connectivity and Structural Defects in Model Hydrogels: A Combined Proton NMR and Monte Carlo Simulation Study. Macromolecules.

[36] Katashima T., Asai M., Urayama K., Chung U.-I., Sakai T. (2014). Mechanical properties of tetra-PEG gels with supercoiled network structure. J. Chem. Phys..

