# Supplementary material for: Evaluation of Mesh Size in Model Polymer Networks Consisting of Tetra-Arm and Linear Poly(ethylene glycol)s
Source: Gels. 2018 May 25;4(2):50. doi: 10.3390/gels4020050 (PMC6209252; doi:10.3390/gels4020050)
Supplement: Supplementary file 1 [file gels-04-00050-s001.pdf]

Supporting Information

Evaluation of Mesh Size in Model Polymer Networks Consisting of Tetra-arm and Linear Poly(ethylene glycol)s

Yui Tsuji[1], Xiang Li[1]*, and Mitsuhiro Shibayama[1]*

[1]Institute for Solid State Physics, The University of Tokyo, Kashiwanoha, Kashiwa, 277-8581, Japan

Estimation of the geometric blob size with tree-like approximation for 2 x 4 gels.

P(F_A^{out}) is the probability of a chain leading to a finite network and p is the reaction efficiency.

Now we consider reaction of A4 and B2.

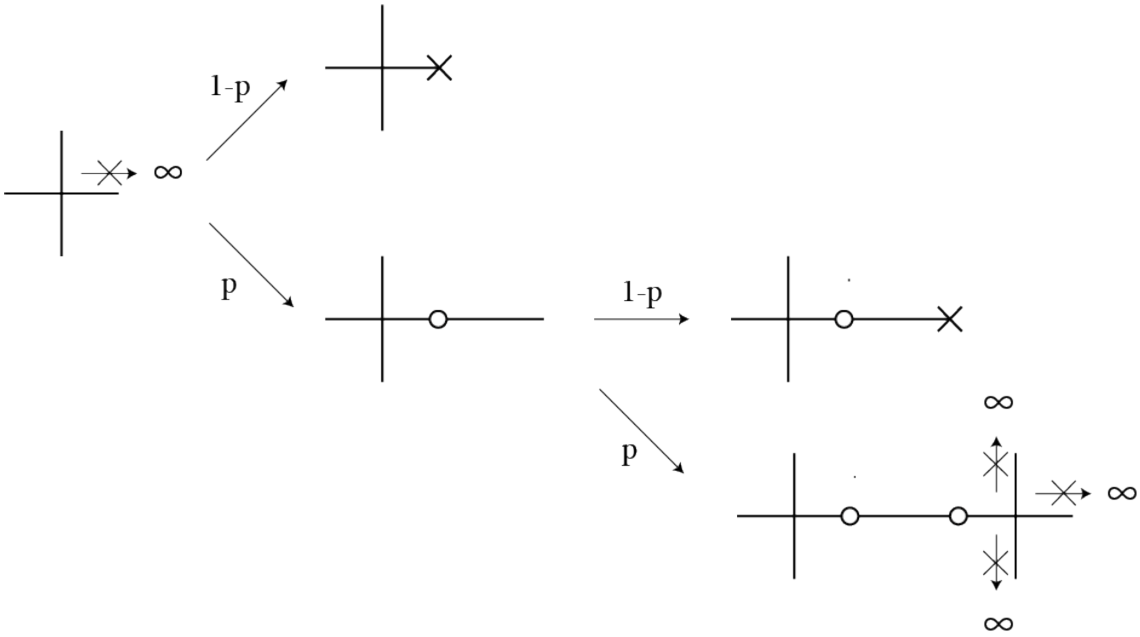

In the first step, there are two cases. One is that the arm of A4 is connected with B2 and the other is that the arm of A4 is not connected with B2. The probability of the latter is 1-p, which is included in P(F_A^{out}). This is because the arm does not lead to an infinite network definitely in this case. In the next step, we consider the former. The former is also separated to two cases.

The one is that A4 is connected with B2 but B2 is not connected. In this case, the probability is p·(1-p). The other is that B2 is connected further with A4. If 3 arms of A4 do not lead to an infinite network, the first root arm is not connected with an infinite network as a result. Then the probability of this case is p·p· $\mathrm{P}(F_A^{out})^3$.

From the above discussion, we can describe $\mathrm{P}(F_A^{out})$ as

$$\mathbf{P}(\boldsymbol{F_A^{out}}) = (\mathbf{1}-\mathbf{p}) + \mathbf{p}\cdot(\mathbf{1}-\mathbf{p}) + \boldsymbol{p}^2\mathbf{P}(\boldsymbol{F_A^{out}})^3 \quad (1)$$

This equation has $\mathrm{P}(F_A^{out}) = 1$ as a solution, but it is not a nontrivial solution. Another solution lies between 0 to 1 from the theory of branching processes. Then the solution of eq(1) was obtained as

$$\mathbf{P}(\boldsymbol{F_A^{out}}) = \left(\frac{\mathbf{1}}{\boldsymbol{p}^2} - \frac{\mathbf{3}}{\mathbf{4}}\right)^{\mathbf{1/2}} - \frac{\mathbf{1}}{\mathbf{2}} \quad (2)$$

$\mathrm{P}(X_f)$ is the probability that A4 becomes f-arm cross-linking point. We can estimate $\mathrm{P}(X_3)$ and $\mathrm{P}(X_4)$ from eq(2).

$$\mathbf{P}(\boldsymbol{X_3}) = {}_{\mathbf{4}}\boldsymbol{C}_{\mathbf{3}}\mathbf{P}(\boldsymbol{F_A^{out}})[\mathbf{1} - \mathbf{P}(\boldsymbol{F_A^{out}})]^{\mathbf{3}} \quad (3)$$

$$\mathbf{P}(\boldsymbol{X_4}) = {}_{\mathbf{4}}\boldsymbol{C}_{\mathbf{4}}[\mathbf{1} - \mathbf{P}(\boldsymbol{F_A^{out}})]^{\mathbf{4}} \quad (4)$$

Then expected value of the number density of cross-linking point (μ) and elastically effective chains (ν).

$$\mu = \Phi\big(\mathrm{P}(X_3) + \mathrm{P}(X_4)\big) \quad (5)$$

$$\nu = \Phi\left(\frac{3}{2}\mathrm{P}(X_3) + \frac{4}{2}\mathrm{P}(X_4)\right) \quad (6)$$

Here, Φ is the number density of A4.
